# Dengue 1 Diversity and Microevolution, French Polynesia 2001–2006: Connection with Epidemiology and Clinics

**DOI:** 10.1371/journal.pntd.0000493

**Published:** 2009-08-04

**Authors:** Elodie Descloux, Van-Mai Cao-Lormeau, Claudine Roche, Xavier De Lamballerie

**Affiliations:** 1 UMR190, Emergence des Pathologies Virales, Université de la Méditerranée and Institut de Recherche pour le Développement, Marseille, France; 2 Laboratoire de Recherche en Virologie Médicale, Institut Louis Malardé, Papeete, Tahiti, French Polynesia; University of California, Irvine, United States of America

## Abstract

**Background:**

Dengue fever (DF) is an emerging infectious disease in the tropics and subtropics. Determinants of DF epidemiology and factors involved in severe cases—dengue haemorrhagic fever (DHF) and dengue shock syndrome (DSS)—remain imperfectly characterized. Since 2000, serotype 1 (DENV-1) has predominated in the South Pacific. The aim of this study was (i) to determine the origin and (ii) to study the evolutionary relationships of DENV-1 viruses that have circulated in French Polynesia (FP) from the severe 2001 outbreak to the recent 2006 epidemic, and (iii) to analyse the viral intra-host genetic diversity according to clinical presentation.

**Methodology/Principal Findings:**

Sequences of 181 envelope gene and 12 complete polyproteins of DENV-1 viruses obtained from human sera in FP during the 2001–2006 period were generated. Phylogenetic analysis showed that all DENV-1 FP strains belonged to genotype IV–“South Pacific” and derived from a single introduction event from South-East Asia followed by a 6-year *in situ* evolution. Although the ratio of nonsynonymous/synonymous substitutions per site indicated strong negative selection, a mutation in the envelope glycoprotein (S222T) appeared in 2002 and was subsequently fixed. It was noted that genetic diversification was very significant during the 2002–2005 period of endemic DENV-1 circulation. For nine DF sera and eight DHF/DSS sera, approximately 40 clones/serum of partial envelope gene were sequenced. Importantly, analysis revealed that the intra-host genetic diversity was significantly lower in severe cases than in classical DF.

**Conclusions/Significance:**

First, this study showed that DENV-1 epidemiology in FP was different from that described in other South-Pacific islands, characterized by a long sustained viral circulation and the absence of new viral introduction over a 6-year period. Second, a significant part of DENV-1 evolution was observed during the endemic period characterized by the rapid fixation of S222T in the envelope protein that may reflect genetic drift or adaptation to the mosquito vector. Third, for the first time, it is suggested that clinical outcome may be correlated with intra-host genetic diversity.

## Author Summary

The molecular characterization of 181 serotype 1 Dengue fever (DENV-1) viruses collected regularly during the 2001–2006 period in French Polynesia (FP) from patients experiencing various clinical presentations revealed that the virus responsible for the severe 2001 outbreak was introduced from South-East Asia, and evolved under an endemic mode until a new epidemic five years later. The dynamics of DENV-1 epidemics in FP did not follow the model of repeated virus introductions described in other South Pacific islands. They were characterized by a long sustained viral circulation and the absence of new viral introduction over a six-year period. Viral genetic variability was not observed only during outbreaks. In contrast with conventional thinking, a significant part of DENV-1 evolution may occur during endemic periods, and may reflect adaptation to the mosquito vector. However, DENV-1 evolution was globally characterized by strong purifying selection pressures leading to genome conservation, like other DENV serotypes and other arboviruses subject to constraints imposed by the host-vector alternating replication of viruses. Severe cases—dengue haemorrhagic fever (DHF) and dengue shock syndrome (DSS)—may be linked to both viral and host factors. For the first time, we report a significant correlation between intra-host viral genetic variability and clinical outcome. Severe cases were characterized by more homogeneous viral populations with lower intra-host genetic variability.

## Introduction

Dengue fever is the most common vector-borne viral disease affecting humans and represents an archetypal emerging infectious disease whose epidemiological landscape has been substantially modified during the past century [1],[2]. Each year, an estimated 100 million people contract dengue fever (DF) in the tropics and subtropics [3] with an increasing incidence of the severe forms, i.e. at least 500,000 cases annually of dengue haemorrhagic fever (DHF) or dengue shock syndrome (DSS).

Dengue virus (DENV) is a member of the genus *Flavivirus* in the family *Flaviviridae*, which includes single-stranded, positive-sense RNA viruses with a genome of approximately 11 kb that encodes three structural proteins (capsid (C), membrane (M), envelope (E)) and seven non structural proteins (NS1, NS2A, NS2B, NS3, NS4A, NS4B, NS5). Four serotypes exist (denoted DENV-1 to DENV-4), the infection by a given serotype conferring a specific and prolonged immunity to that serotype [4]. The factors that lead to severe infections are debated, and may include both viral factors (e.g., differences in strain virulence [5]–[7]), host immune factors such as antibody-dependent enhancement, cell-mediated immunity [8]–[10] and antigenic mimicry [11].

French Polynesia (FP) which comprises more than one hundred South Pacific islands, has experienced a large number of dengue fever epidemics involving all four serotypes (DENV-1 in 1944, 1975–76, 1988–89, 2001; DENV-2 in 1971, 1996–97; DENV-3 in 1964, 1969, 1989–90; and DENV-4 in 1979, 1985) [12]–[16]. Approximately 260,000 inhabitants live in FP, mostly in the Society Archipelago and particularly in Tahiti but importantly a large number of tourists from Asia, Central and South America, and other Pacific islands visit FP annually [17] possibly inadvertently introducing new DENV strains.

Since 2000, DENV-1 has been the predominant serotype in the Pacific region [18] causing successive outbreaks (Palau in 2000; FP, Samoa, Hawaii and Easter island in 2001; Cook and Solomon islands in 2002; Wallis, Futuna and New Caledonia in 2002–2003). After the severe DENV-1 outbreak which caused nearly 33,800 cases in 2001 [15], FP experienced a period of low-level transmission from 2002 to 2005, followed by a new epidemic in 2006 [19].

In this study, we performed an analysis of the E-gene sequence of 181 DENV-1 viruses and the nearly complete coding sequence of 12 DENV-1 viruses collected over a 6-year period from patients experiencing various clinical presentations in the five FP archipelagos. In addition, we performed a comprehensive comparative analysis of intra-host viral genetic diversity in 16 patients. This study enabled us to predict the precise geographic origin and evolutionary relationships, during both endemic and epidemic periods, of the DENV-1 isolates that circulated in FP from the severe 2001 outbreak to the recent 2006 epidemic. Original patterns of intra-host genetic diversity were also identified in association with the clinical severity of infection.

## Methods

### Specimen data

**Figure 1:**
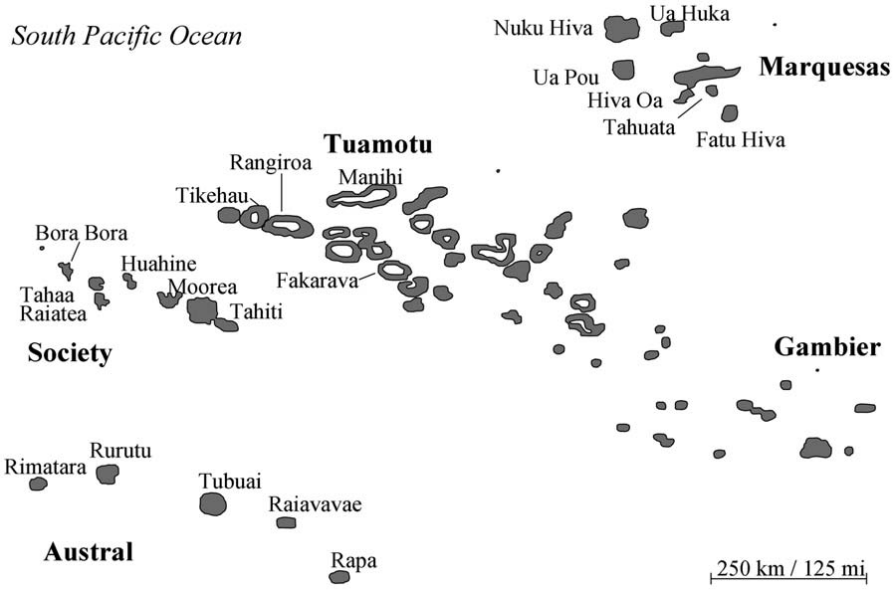
Map of French Polynesia (FP). Sampling of DENV-1 sera was conducted in the five FP archipelagos: Society, Tuamotu, Gambier, Austral and Marquesas.

We analyzed serum samples from 181 DENV-1 infected patients from FP. Sampling was conducted in the five FP archipelagos (Figure 1): Society (Windward and Leeward islands), Tuamotu, Gambier, Austral and Marquesas, from January 2001 to December 2006 (Table 1). The study period included the 2001 and 2006 DENV-1 outbreaks, separated by four years of low-level transmission (2002–2005). From a total of 181 cases, 152 patients experienced DF, 19 DHF and ten DSS with one death. Dengue disease severity was graded according to the World Health Organization (WHO) classification guidelines [20]. The time of serum collection relative to infection ranged from one to six days in documented cases. All human sera analyzed in this study had been preserved at −80°C at the Institut Louis Malardé (Tahiti, FP).

**Table 1:** Geographical and clinical characteristics of the 181 DENV-1 sera used for E-gene sequence analysis.

Geographical origin							Clinical presentation^a^	
	Society archipelago		Austral archipelago	Tuamotu archipelago	Marquesas archipelago	Gambier archipelago	DF	severe infection (DHF, DSS, death)
	Windward islands (Tahiti, Moorea)	Leeward islands (Bora Bora, Raiatea, Tahaa, Huahine)						
**2001**	Tahiti 19	Bora Bora 6	1	3	-	1	28	12 DHF
n = 49	Moorea 5	Raiatea 11						9 DSS (1 death)
		Tahaa 1						
		Huahine 2						
**2002**	Tahiti 26	Bora Bora 1	3	1	2	-	39	3 DHF
n = 43	Moorea 7	Raiatea 2						1 DSS
		Huahine 1						
**2003**	Tahiti 14	-	-	-	-	-	15	-
n = 15	Moorea 1							
**2004**	Tahiti 3	Raiatea 2	-	-	-	-	9	-
n = 9	Moorea 4							
**2005**	Tahiti 6	-	-	-	-	-	15	1 DHF
n = 16	Moorea 10							
**2006**	Tahiti 19	Bora Bora 7	3	1	-	-	46	3 DHF
n = 49	Moorea 8	Raiatea 7						
		Tahaa 2						
		Huahine 2						

a DF = dengue fever , DHF = dengue hemorrhagic fever , DSS = dengue shock syndrome.

### Ethics statement

All samples were obtained from sera initially sampled for diagnostic purpose, and archived at the Institut Louis Malardé (Tahiti, FP). The use of biological samples and the collection of information were performed with the authorization of the “Direction des affaires juridiques et des droits des patients, Centre Hospitalier Territorial de Polynésie Française (Tahiti)” and in accordance with French regulations.

### Molecular characterization

Virus RNA was extracted from acute-phase sera of DENV-1 infected patients using the QIAamp Viral RNA Mini Kit (Qiagen) according to manufacturer's instructions.

DENV-1 sequences were retrieved from public databases and used to design oligonucleotide primers for reverse transcription-polymerase chain reaction (RT-PCR) amplification and sequencing of FP viruses.

Genetic characterization of the E-gene was conducted using the Qiagen OneStep RT-PCR kit together with primers E1F-E4R, followed by a nested PCR using primers E2F-E3R (Table S1) to produce a 1,759 nt fragment including the complete E-gene (1,485 nt) which was subsequently sequenced directly using amplification primers.

For characterization of full-length coding sequences, 12 overlapping cDNA fragments were generated by RT-PCR using 12 sets of oligonucleotide primers (Table S1). Fragments C1, C3–C8, C10 and C11 were obtained using the same one-step RT-PCR protocol as described above. Fragments C2, C9 and C12 were synthesized using a two-step protocol: cDNA was generated using a mixture of random hexaprimers (RT Taqman Applied Biosystems) followed by PCR amplification using *Taq* Polymerase (Invitrogen). Sequencing using amplification primers resulted in the characterization of a 10,075 nt sequence.

For the analysis of intra-host genetic diversity, the Qiagen OneStep RT-PCR kit was used together with primers Q1F-Q1R (Table S1) to produce a 758 nt fragment within the E-gene, which was subsequently purified using the QIAquick PCR Purification Kit, ligated into the cloning vector pCR 2.1 and transformed into TOP10 competent cells, according to the manufacturer's protocol (TA Cloning, Invitrogen). Approximately 40 clones per serum were generated and sequenced using the T7 promoter primer (5′-CCCTATAGTGAGTCGTATTA-3′). To estimate the error rates of our amplification system, we carried out a control experiment using a fully sequenced clone of the 758 nt fragment. Serial dilutions were produced and the last dilution providing a clear positive signal was used as a control. It was submitted to one-step RT-PCR amplification and clones (n = 90) were characterized under identical conditions as viral RNA extracted directly from acute phase DENV-1 patient sera. In order to evaluate the influence of viral load in DENV-1 genetic diversity within patients, viral RNA was quantified by real-time RT-PCR, as described previously [21] in 11 of the 17 analyzed sera corresponding to five DHF/DSS cases and five DF cases (including sequential blood samples for one patient: 47.2002 and 49.2002).

### Phylogeny and sequence analysis

Sequence data from sequencing reactions were combined for analysis and edited using the Sequencher 4.7 software (Gene Codes Corporation). Nucleotide sequences used for phylogenetic analyses were aligned using Clustal W [22], and then imported into the MEGA 3.1 package [23]. Nucleotide genetic distances were calculated using the Kimura 2 algorithm [24] and Neighbor-Joining was used for phylogenetic reconstructions. Robustness of phylogenetic trees was assessed using bootstrap resampling analysis (1000 replications). Supplementary maximum likelihood phylogenetic analyses were performed using the Bayesian method available in MrBayes v3.1.2 [25] with a minimum of ten million generations and a burnin of 10%. Stationary was assessed at effective sample size (ESS)>400 using Tracer v1.4.1 (part of the BEAST package [26]).”

Phylogenetic analysis of E-gene sequences was conducted using a sample of 240 DENV-1 sequences. This included 181 FP sequences generated in this study together with three sequences of viruses that were previously characterized during the 1988–89 and the 2001 DENV-1 outbreaks in FP [27]: D1.French Polynesia/89, GenBank accession number AY630408; D1.French Polynesia/01, GenBank accession numbers AY630407 and AB111070. These FP sequences were combined with a sample of 56 viruses representing the global genetic variability of DENV-1 available from GenBank. In addition, we conducted a phylogenetic analysis based on the complete coding regions of 41 DENV-1 strains isolated worldwide (available from GenBank) and the corresponding sequences of 12 FP strains characterized in this study.

Differences in nucleotide and protein sequences were analyzed and compared according to the geographical origin, the sampling period and the clinical presentation. The extent of sequence divergence was evaluated using the pairwise distance among the nucleotide sequences (π nt) and the amino acid sequences (π aa). The mean ratio of nonsynonymous (d_N_) to synonymous (d_S_) substitutions per site was estimated using the pairwise method of Nei and Gojobori [28] as implemented in the MEGA 3.1 package.

For the analysis of intra-host genetic diversity, the sequence of each clone was compared to all other clones for each human serum. The percentage of variable nucleotide sites (number of variable nt sites/number of nt sites), of nucleotide mutations (number of nt mutation/number of nt sequenced), and of mutant clones (number of clones with mutation/total number of clones) was calculated, as well as the π nt, π aa, d_N_, d_S_ and d_N_/d_S_ parameters. Results were then compared according to the clinical presentation of dengue infection.

To explore the selection pressures acting on DENV-1 at different levels of viral evolution, distinct datasets were analyzed as follows: (i) “FP intra-host” dataset: this group included 17 series of cloned sequences obtained from 16 patients infected with DENV-1 (eight DF, eight DHF/DSS) in FP between 2001 and 2006. For one patient (DF - Moorea, Windward islands, Society archipelago - December 2002) two series of clones were produced from sequential blood samples obtained at day one and day four of the disease ; (ii) “FP inter-host” dataset: this group included the 181 sequences generated in FP between 2001 and 2006 (this study) ; (iii) “genotype IV inter-host” dataset: this included 26 sequences representing the genetic diversity of the “South Pacific” genotype; (iv) “serotype 1 inter-host” dataset: this included 59 sequences that reflect the worldwide diversity of DENV-1 isolates. For each dataset, the same parameters (percentage of variable nucleotide sites, π nt, d_S_, d_N_, d_N_/d_S_) were analyzed.

### Statistical analysis

All statistical analyses were performed using the R software package (R development Core Team version 2.6.0). Categorical and binary variables were compared using a Fisher's exact test. A Mann-Withney test was used for continuous variables (p values below 0.05 were considered to indicate statistical significance).

To evaluate differences between endemic and epidemic periods, a panel of 176 samples collected between March 2001 and December 2006 was analyzed (five samples collected in February 2001 before the beginning of the 2001 outbreak were excluded). To assess differences in nucleotide sequences, we compared the matrix of pairwise distances obtained for 93 sequences of DENV-1 viruses sampled during the 2002–2005 endemic period and the matrix of pairwise distances obtained for 83 sequences of DENV-1 viruses sampled during epidemics (i.e. a first matrix obtained from the 42 sequences related to the 2001 FP outbreak, combined with a second matrix from the 41 sequences related to the 2006 FP outbreak).

For the analysis of intra-host genetic variability according to the clinical severity of dengue infection, we compared the percentage of variable nucleotide sites, the percentage of nucleotide mutation, the percentage of mutant clones, the average pairwise distance (π nt) and the mean d_N_, d_S_, d_N_/d_S_ ratio obtained for each group of clones in nine DF sera (including two sequential blood samples for one patient) versus eight DHF or DSS sera.

## Results

### Phylogenetic analysis

**Figure 2:**
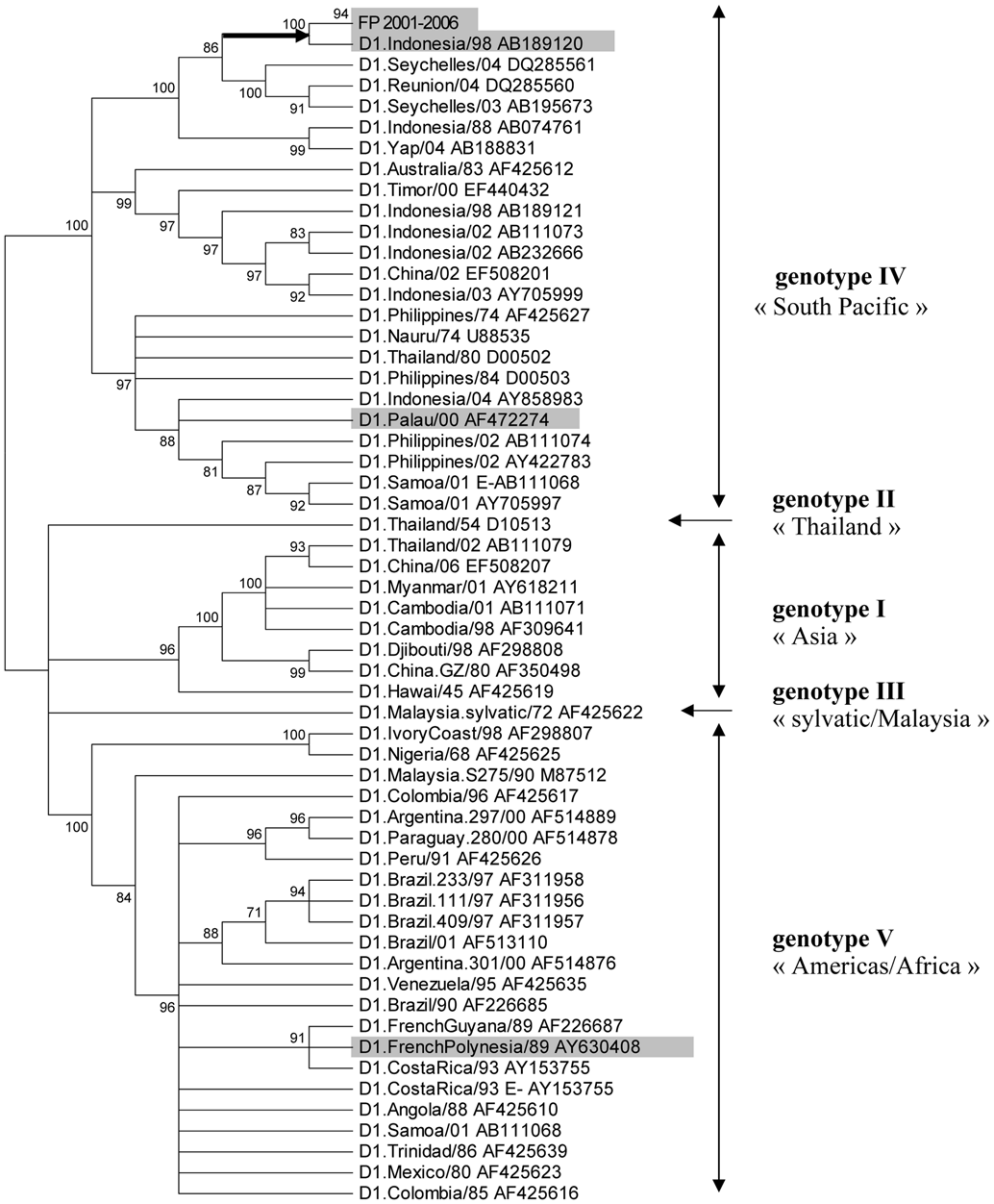
Phylogenetic tree based on 240 DENV-1 nucleotide sequences of 1,759 bp including the E-gene (Neighbor-Joining method, Kimura 2 algorithm). The 181 sequences generated in FP are condensed in the branch named “FP 2001–2006”. Taxon names of GenBank sequences correspond to D1.country/last two digits of year of isolation and GenBank accession number. In this condensed tree, branch length is not proportional to genetic distance. Numbers on branches represent bootstrap support for each branch. Five DENV-1 genotypes were identified. The validity of these genotypes, in particular genotype II “Thailand” and genotype III “sylvatic/Malaysia”, is supported by previous phylogenetic analyses based on maximum likelihood method [5],[29].”

Phylogenetic analysis of 240 E-gene nucleotide sequences (including the 181 FP sequences generated in this study) allowed the identification of DENV-1 genotypes I to V [29] previously defined “Asia”, “Thailand”, “sylvatic/Malaysia”, “South Pacific”, and “Americas/Africa” genotypes, respectively, according to their apparent geographic origin (see Figure 2) [5].

The phylogenetic reconstruction based on E-gene nucleotide sequences showed that all DENV-1 strains that circulated in FP between 2001 and 2006 fall into genotype IV – “South Pacific” (Figures 2 and S1). This genotype also includes DENV-1 viruses originating from other locations in the Pacific (Australia, Malaysia, Philippines, Palau, Yap, Nauru, Samoa, Hawaii), from South-East Asia (Thailand, Myanmar, China, Indonesia, Timor), and from the Indian Ocean (Seychelles, Reunion) between 1974 and 2006. According to a previous study [27], the D1.French Polynesia/89 strain isolated during the 1988–89 DENV-1 epidemic (preceding the 2001 outbreak) belonged to a different genotype (genotype V “Americas/Africa”) and was very close to D1.French Guyana/89.

**Figure 3:**
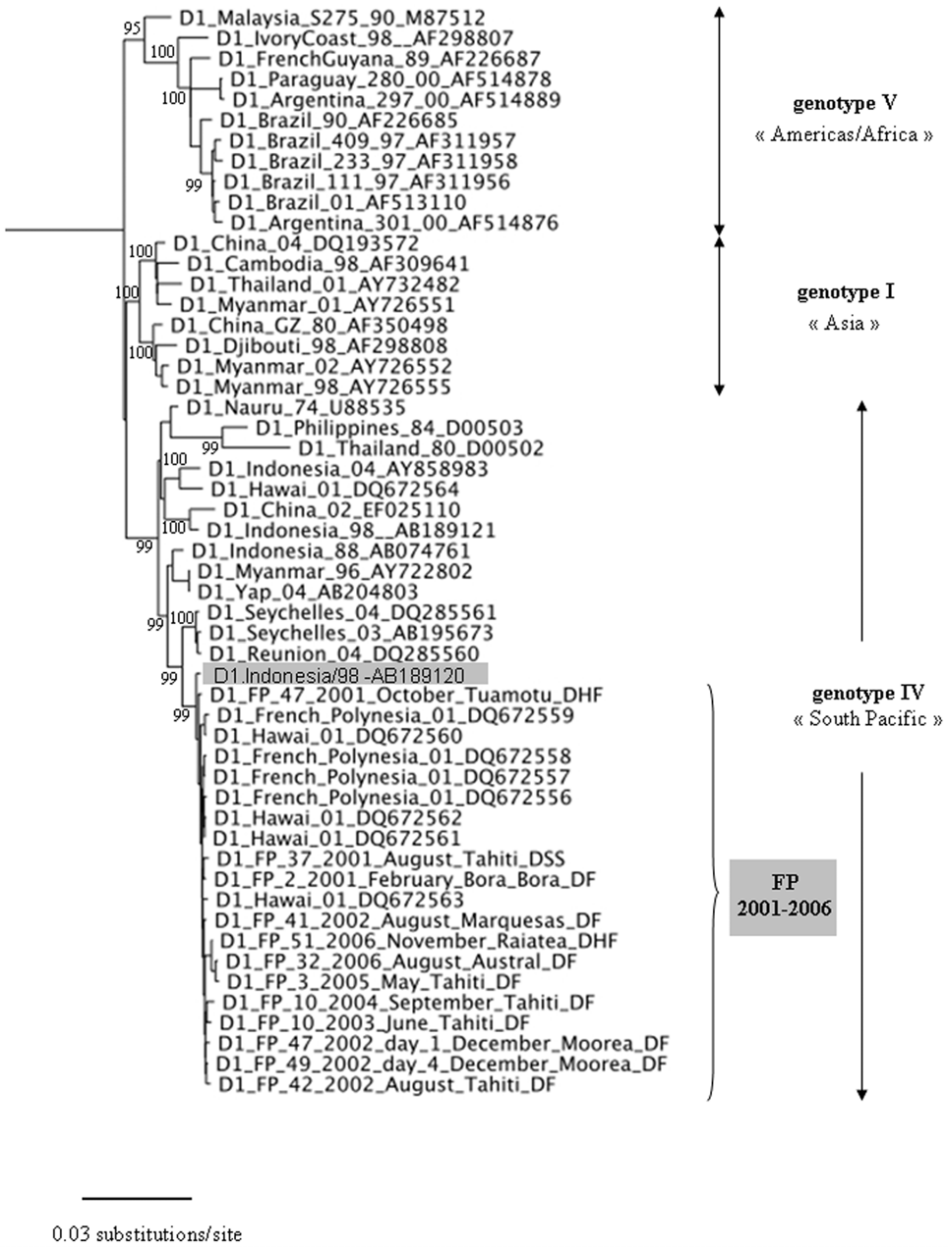
Phylogenetic tree based on 53 DENV-1 complete genome amino acid sequences (maximum likelihood phylogenetic analysis using Bayesian method). Taxon names of FP sequences correspond to D1_FP_sample number_year_month_geographical origin_clinical presentation. Taxon names of GenBank sequences correspond to D1_country_last two digits of year of isolation_GenBank accession number. Posterior probabilities (percent) are shown for values >80 only. All horizontal branch lengths are drawn to a scale of substitutions per site. The tree was rooted using a DENV-3 strain (D3_SriLanka_00_NC001475, not shown for purposes of clarity only).

DENV-1 strains recovered in FP during the severe 2001 epidemic shared a common ancestor with D1.Indonesia/98, a strain isolated in 1998 in a patient from Indonesia (Figures 2 and S1). However, they were more distantly related to D1.Palau/00, a strain isolated in Palau (Micronesia), the first island affected by DENV-1 in the Pacific Ocean in the 2000's [30]. The D1.Palau/00 strain was found to be more closely related to strains isolated during DENV-1 epidemics in the Philippines or Samoa islands in 2001 and 2002. Altogether, these results strongly suggest that DENV-1 that circulated in FP in 2001 originated from Indonesia rather than from Palau. This finding is further supported by phylogenetic analysis of 53 complete polyprotein sequences (Figures 3 and S2). In addition, phylogenetic analysis showed that most DENV-1 strains recovered during the 2001 outbreak in Hawaii clustered in the same lineage as FP 2001 strains (Figures 3 and S2), suggesting a Polynesian origin of the DENV-1 epidemic that occurred in Hawaii in 2001 [31].

A more detailed phylogenetic analysis of FP 2001–2006 sequences suggested that all FP viruses characterized in this study derived from a common ancestor, i.e. originated from a single introduction event in FP followed by a 6-year *in situ* evolution (Figures 3, S1 and S2). Notably, the observed evolutionary pattern globally follows the chronology of viral spread rather than the geographical origin of viruses or the clinical severity of cases. However, a subgroup comprising seven 2006 strains (10-33-49-50-51-52-57.2006) was found to include viruses originating from Moorea, Raiatea, Tahaa, or the Austral archipelago, but did not include any of the 19 strains that infected patients in Tahiti in 2006 (Figure S1).

### Analysis of sequence divergence

The polyprotein sequences of 12 FP 2001–2006 DENV-1 viruses were studied. Nonsynonymous mutations were observed in all genes, except NS4A. Amongst them, four mutations located in the E, NS4B, and NS5 genes have been fixed during viral evolution (Table 2).

**Table 2:** Analysis of polyprotein sequences of 12 DENV-1 viruses recovered in FP between 2001 and 2006.

DENV-1 proteins	No. of variable aa sites/No. of aa analysed (%)		aa changes (position^a^)		Characteristics of samples with aa changes
					Serum number, clinical presentation^b^, geographical origin
Polyprotein	23/3358	(0.68%)			
Capsid (C)	2/114	(1.75%)	G→S	(9)	32.2006 DF Austral, 51.2006 DHF Raiatea
			V→I	(26)	47.2002 DF Moorea
Membrane (M)	1/166	(0.60%)	S→Y	(226)	42.2002 DF Tahiti
Envelope (E)	3/495	(0.61%)	S→F	(418)	41.2002 DF Marquesas
			S→T	(502)	all strains since August 2002 (n = 8)^c^
			K→R	(643)	51.2006 DHF Raiatea
NS1	3/352	(0.85%)	V→I	(868)	47.2001 DHF Tuamotu
			S→G	(892)	3.2005 DF Tahiti
			N→H	(1068)	32.2006 DF Austral
NS2A	3/218	(1.38%)	L→M	(1204)	49.2002 DF Moorea
			L→V	(1204)	10.2003 DF Tahiti
			A→T	(1215)	10.2004 DF Tahiti
			V→M	(1238)	10.2004 DF Tahiti
NS2B	1/130	(0.77%)	H→Y	(1467)	47.2002 DF Moorea
NS3	1/619	(0.16%)	E→D	(2056)	3.2005 DF Tahiti
NS4A	0/150	(0.00%)			
NS4B	4/249	(1.61%)	A→T	(2262)	all strains since May 2005 (n = 3)^c^
			V→G	(2661)	10.2004 DF Tahiti
			E→K	(2662)	10.2004 DF Tahiti
			P→H	(2663)	10.2004 DF Tahiti
NS5	5/899	(0.56%)	T→I	(2727)	10.2004 DF Tahiti
			W→L	(2924)	10.2004 DF Tahiti
			D→E	(3037)	all strains since August 2002 (n = 9)^c^
			T→I	(3144)	all strains since May 2005 (n = 3)^c^
			A→T	(3349)	51.2006 DHF Raiatea

a The numbering of amino acid (aa) positions was based on the numbering of the complete coding region (3392 aa) of DENV-1.

b DF = dengue fever, DHF = dengue hemorrhagic fever, DSS = dengue shock syndrome.

c Four mutations have been fixed in the E, NS4B and NS5 genes.

Of note, most aa changes occurred during the 2002–2005 endemic period.

**Figure 4:**
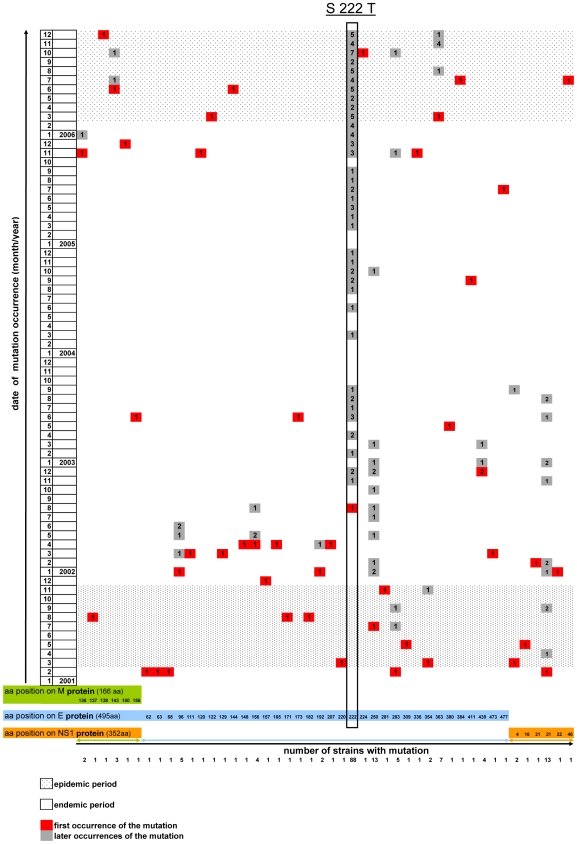
Variability of 181 protein sequences of DENV-1 during epidemic and endemic periods in FP from 2001 to 2006. Ten amino acid changes newly occurred during the 2001 outbreak, 23 during the 2002–2005 endemic period, and eight during the 2006 outbreak. S222T appeared in August 2002 and was subsequently fixed by viral evolution.

Additional analyses were conducted on a 1,759 nt region that encompassed the complete E-gene for 181 FP 2001–2006 sequences (Table 3). A panel of 93 samples collected during the 2002–2005 endemic years and 83 samples collected during the 2001 and the 2006 outbreaks were analyzed. Synonymous and nonsynonymous mutations were observed both during epidemic and endemic periods. The number of variable sites was found to be significantly higher during endemic period than during epidemics. The nucleotide sequence divergence (π nt) was also higher during endemic than during epidemic periods (p<0.001). When focusing on the first appearance of amino acid changes, 56% occurred during the 2002–2005 endemic period (most of them during the 2002 post-epidemic year) whereas 24% and 20% occurred during the 2001 and 2006 epidemics, respectively (Figure 4). The number of new amino acid changes decreased from 2002 to 2004 and slightly increased in 2005.

**Table 3:** Genetic diversity of DENV-1 at different levels and at different times of viral evolutionary divergence based on a 1,759 nt fragment including the E-gene.

Dataset^a^	No. of variable nt sites/No. of nt analysed	No. of variable aa sites/No. of aa analysed	π nt^b^	
	%	%	mean	range
**Serotype 1**	537/1759	92/586		
59 sequences	30.5%	15.7%	6.5%	0–10%
**Genotype IV**	338/1759	47/586		
26 sequences	19.2%	8.0%	4.3%	0–9.1%
**FP 2001–2006**	128/1759	47/586		
181 sequences	7.3%	8.0%	0.4%	0–1%
**FP 2001 epidemic period**	34/1759	13/586		
42 sequences^c^	1.9%	2.2%	0.1%	0–0.3%
**FP 2002–2005 endemic period** d	78/1759	27/586		
93 sequences	4.4%	4.6%	0.3%	0–0.9%
**FP 2006 epidemic period**	28/1759	10/586		
41 sequences	1.6%	1.7%	0.2%	0–0.5%

a Sequences in datasets “Serotype 1”, “Genotype IV” and “FP 2001–2006” were also used for phylogenetic reconstructions (Figures 2, 3, S1, S2).

b The pairwise distances were calculated among the nucleotide sequences in each dataset (π nt).

c Five samples collected in February 2001 before the beginning of the 2001 outbreak were excluded from the comparative analysis of DENV-1 evolution during the endemic and epidemic periods (a total of 176 samples collected between March 2001 and December 2006 was analyzed).

d Results were significantly different between endemic and epidemic periods (see Methods for details of statistical analysis).

The most frequent mutation (nt T664A, E-gene numbering), observed in 88 of 181 strains, corresponded to a nonsynonymous substitution (aa S222T). Figure 4 clearly shows that this mutation was not present in 2001; it appeared in August 2002 (endemic period) and was rapidly fixed (9% of sequenced strains in 2002, 67% in 2003, 100% in 2004, 2005 and 2006). Another interesting event was the occurrence of the K363R mutation in domain III of the envelope protein in a cluster of seven 2006 strains (see above). All strains in this subgroup were isolated in patients with DF or DHF in Moorea, Raiatea, Tahaa, or the Austral archipelago, whereas this mutation was absent in all strains that infected patients in Tahiti in 2006.

### Intra-host genetic diversity of DENV-1 and the estimation of selection pressures

To examine the extent of genetic diversity of DENV-1 *in vivo* at the intra-host level, we sequenced 662 clones corresponding to partial E-genes of DENV-1 populations from 16 human sera at a single time point during acute infection. For one DF patient, clones corresponding to sequential samples (day one and day four of the symptoms) were sequenced and compared. Approximately 40 clones from each sample were analyzed, and the results are summarized in Table 4.

**Table 4:** Intra-host genetic diversity analysis of DENV-1 populations from 17 sera of patients infected in FP between 2001 and 2006.

DENV-1 samples characteristics serum number clinical presentation^a^, sex/age patient, geographical origin, sample date (month/year)	No. of clones	% of mutant clones	% of variable nt sites	% of nt mutations	π nt	π aa	d_N_	d_S_	d_N_/d_S_
**serum 2.2001**	41	**61%**	6%	**0.17%**	**0.3%**	0.7%	0.003	0.005	0.600
DF. F/7 years									
Bora Bora 02/2001									
**serum 7.2001**	53	**77%**	8%	**0.18%**	**0.4%**	0.7%	0.003	0.005	0.600
DHF. M/30 years									
Tahiti 03/2001									
**serum 14.2001**	39	**56%**	4%	**0.10%**	**0.2%**	0.4%	0.002	0.002	1.000
DHF. F/55 years									
Raiatea 04/2001									
**serum 26.2001**	50	**62%**	8%	**0.16%**	**0.3%**	0.7%	0.003	0.003	1.000
DSS. F/12 years									
Tahiti 06/2001									
**serum 35.2001**	48	**77%**	8%	**0.20%**	**0.4%**	0.8%	0.004	0.005	0.800
DSS. M/6 years									
Tahiti 08/2001									
**serum 37.2001**	39	**49%**	4%	**0.11%**	**0.2%**	0.3%	0.001	0.005	0.200
DSS. M/6 years									
Tahiti 08/2001									
**serum 47.2001**	24	**88%**	2%	**0.19%**	**0.3%**	0.5%	0.002	0.008	0.250
DHF. F/11 years									
Tuamotu 10/2001									
**serum 41.2002**	31	**48%**	9%	**0.33%**	**0.7%**	1.4%	0.006	0.008	0.750
DF. F/13 years									
Marquesas 08/2002									
**serum 42.2002**	36	**75%**	10%	**0.37%**	**0.8%**	1.5%	0.007	0.010	0.700
DF. F/13 years									
Tahiti 08/2002									
**serum 47.2002**	34	**82%**	8%	**0.34%**	**0.7%**	1.2%	0.005	0.011	0.455
DF. M/6 years									
Moorea 12/2002 day 1^b^									
**serum 49.2002**	38	**76%**	9%	**0.29%**	**0.6%**	1.1%	0.005	0.009	0.556
DF. M/6 years									
Moorea 12/2002 day 4^b^									
**serum 10.2003**	35	**77%**	11%	**0.57%**	**1.1%**	2.6%	0.011	0.009	1.222
DF. F/43 years									
Tahiti 06/2003									
**serum 10.2004**	34	**88%**	10%	**0.44%**	**0.8%**	1.5%	0.007	0.012	0.583
DF. M/51 years									
Tahiti 09/2004									
**serum 3.2005**	44	**59%**	4%	**0.15%**	**0.3%**	0.4%	0.002	0.005	0.400
DF. M/38 years									
Tahiti 05/2005									
**serum 14.2006**	49	**92%**	8%	**0.26%**	**0.4%**	0.8%	0.003	0.007	0.429
DHF. F/43 years									
Tahiti 03/2006									
**serum 32.2006**	28	**54%**	4%	**0.19%**	**0.4%**	0.7%	0.003	0.006	0.500
DF. M/14 years									
Austral 08/2006									
**serum 51.2006**	39	**54%**	5%	**0.15%**	**0.3%**	0.6%	0.002	0.004	0.500
DHF. M/14 years									
Raiatea 11/2006									
**total 17 sera**	662								
average	39	**69%**	**7%**	**0.25%**	**0.5%**	0.9%	0.004	0.007	0.620
**9 DF sera**	321								
average	36	**69%**	**8%**	**0.32%** c	**0.6%** c	1.2%^c^	0.005^c^	0.008^c^	0.641
**8 DHF/DSS sera**	341								
average	43	**69%**	**6%**	**0.17%** c	**0.3%** c	0.6%^c^	0.003^c^	0.005^c^	0.597

Each clone sequence was compared to other clone sequences for each serum sample.

The percentage of variable nucleotide (nt) sites was the number of variable nt sites ×100 divided by the number of nt analysed (758 nt).

The percentage of nucleotide (nt) mutations was the number of nt mutations ×100 divided by the number of nt sequenced for each serum sample.

The average pairwise distance was calculated among the nucleotide (π nt) and amino acid (π aa) sequences in each serum.

The mean ratio of non-synonymous (d_N_) and synonymous (d_S_) substitutions per site were estimated using the pairwise method of Nei and Gojobori.

a DF = dengue fever, DHF = dengue haemorraghic fever, DSS = dengue shock syndrome.

b Strains 47.2002 and 49.2002 corresponded to sequential samples from the same patient (day 1 and day 4 of the symptoms).

Overall, the time of serum collection relative to infection that do not appear to be a critical parameter for the study of DENV intra-host genetic diversity [42],[43] ranged from one to six days in documented cases.

c Differences between DF and DHF sera were statistically significant (p<0.05, see Methods for details of statistical analysis).

We carried out a control experiment to evaluate the sequence variation due to *in vitro* polymerase errors (see Methods). Among 90 clones of the 758 nt fragment studied within the E-gene, 55 nucleotide substitutions were found, corresponding to an error frequency of 0.10% or 24×10−6 changes/nt/PCR cycle. This result was significantly lower than the mean levels of intra-host diversity (percentages of nucleotide mutations) observed in our samples (0.25% or 60×10−6 changes/nt/PCR cycle, p<0.001).

In the 17 human sera studied, a high proportion of mutant clones was observed (mean 69%) with no significant difference in terms of clinical presentation, endemic or epidemic period, and time of sampling: analysis of sequential blood samples indicated that DENV-1 viraemia comprised a genetically heterogeneous mixture of variants that were present at the time of first appearance of the symptoms.

Mutations occurred in 15 (2%) to 81 (11%) sites of the 758 nucleotides sequenced. The proportion of nonsynonymous mutations was very high in each group of clones (63% on average). Most mutations were observed only once. However, identical mutations were sometimes observed in several clones from the same serum and/or in different sera. For instance, E269K was observed in 16 clones (strains 41.2002, 47.2002, 49.2002, 10.2003, and 10.2004) and E309K in 45 clones (strains 37.2001, 41.2002, 42.2002, 47.2002, 49.2002, 10.2003, 10.2004, and 32.2006). They were present simultaneously in 14 clones (strains 41.2002, 47.2002, 49.2002, 10.2003 and 10.2004). Of note, the mutation S222T was recovered in all clones of the 42.2002 strain, the first DENV-1 strain that expressed the mutation in August 2002 in Tahiti, and it was absent in all clones tested from sera of patients who were infected previously.

Overall, clones with in-frame stop codons were identified in 9 of the 17 sera studied, with a frequency ranging from 0% to 12% (strain 47.2002). Over a total of 662 clones studied, 18 included stop codons (3%) at aa positions 202, 206, 211, 233, 248, 271, 284, 323, 328, 340 (2 clones/1 virus), 370 (3 clones/2 viruses), 420, 426 and 434 (2 clones/1virus) in the E-protein (495 aa). They occurred in DF, DHF and DSS, during outbreak and endemic periods.

A comparative analysis of DENV-1 intra-host genetic diversity was conducted in 16 patients who had experienced infections of different severity (eight DF versus eight DHF and DSS) in FP between 2001 and 2006 (Table 5). The percentage of nucleotide mutations (number of nt changes/number of nt sequenced) was significantly lower in severe (DHF and DSS) clinical presentations (mean 0.17%, range 0.10%–0.26%) than in classical forms (DF) of dengue infection (mean 0.32%, range 0.15%–0.57%, p = 0.015). Moreover, the mean sequence divergence was found to be lower in severe cases than in DF cases (p = 0.014 for π nt, p = 0.025 for π aa). Despite a similar proportion of mutant clones in DF and severe cases (69%), d_N_ and d_S_ were significantly lower in the latter cases (p = 0.014 and p = 0.011, respectively). When error frequencies calculated in our control experiment were subtracted from the results obtained for DF, DHF and DSS clones, differences between severe and classical cases remained significant (data not shown). Altogether, these findings indicate that the level of intra-host genetic diversity is lower in severe presentations than in classical forms of DENV-1 infection. In order to evaluate the influence of viral load in DENV-1 genetic diversity within patients, viral RNA was quantified in five DHF/DSS sera and five DF sera (including sequential serum samples for one patient: 47.2002 and 49.2002). Ct (cycle threshold) levels indicated comparable viral loads in both DF and DHF/DSS serum samples (mean Ct = 29.5 and 28.3, respectively). No correlation was found between the level of intra-host genetic diversity and viral load (range 0.12*10^5^–4.5*10^5^, mean 1.10*10^5^ RNA copies/µL): linear regression analysis showed that the percentage of nt mutations and π nt were not correlated with viral load (p = 0.51 and 0.61, respectively). Moreover, in these serum samples, viral loads were not significantly different in severe cases than in DF cases (p = 0.31, Mann-Withney test).

**Table 5:** Analysis of genetic variability in DENV-1 at different levels of evolutionary divergence based on a 758 nt fragment in the E-gene.

Dataset	No. of variable nt sites/No. of nt analysed %	No. of variable aa sites/No. of aa analysed %	π nt	d_N_	d_S_	d_N_/d_S_
**INTER-HOST** a						
**Serotype 1**	245/758	25/252				
59 sequences	32.3%	9.9%	**6.0%**	0.010	0.223	**0.045**
**Genotype IV**	135/758	25/252				
26 sequences	17.8%	9.9%	**3.8%**	0.008	0.138	**0.058**
**FP 2001–2006**	47/758	17/252				
181 sequences	6.2%	6.7%	**0.3%**	0.002	0.006	**0.333**
**INTRA-HOST**						
**FP 2001–2006**	53/758	34/252				
662 clones (17 sera) sequences^b^	7.0%	13.5%	**0.5%**	0.004	0.007	**0.620**

a Sequences in datasets “Serotype 1”, “Genotype IV” and “FP 2001–2006” were also used for phylogenetic reconstructions (Figures 2, 3, S1, S2).

b The average of the results obtained for each individual (Table 5) was used for intra-host data analysis.

The average pairwise distance was calculated among the nucleotide sequences in each dataset (π nt).

The mean ratio of synonymous (d_S_) and non synonymous mutations per site (d_N_) were estimated using the pairwise method of Nei and Gojobori.

ABBREVIATIONS

aa: amino acid

DENV: dengue virus

DF: dengue fever

DHF: dengue haemorrhagic fever

DSS: dengue shock syndrome

d_N_: ratio of nonsynonymous substitutions per site

d_S_: ratio of synonymous substitutions per site

FP: French Polynesia

nt:nucleotide

RT-PCR: reverse transcription-polymerase chain reaction

π nt: nucleotide sequence divergence (pairwise distance)

Finally, the mode of evolution of DENV-1 in FP was investigated by analysing the mean ratio of nonsynonymous to synonymous substitutions per site (d_N_/d_S_) in our different dataset: d_N_/d_S_ was 0.100 for complete genome sequences, and 0.091 for E gene sequences, indicating (d_N_/d_S_<1) a strong negative (purifying) selection pressure [32]. This was confirmed by the study of the genetic variability at different levels of evolutionary divergence, i.e. in the four datasets: “FP intra-host”, “FP inter-host”, “genotype IV inter-host”, and “serotype 1 inter-host” (Table 5). Within the group of FP viruses, the genetic variability of DENV-1 was higher within hosts than between hosts, as indicated by π nt, and d_N_/d_S_ values which were higher in the intra-host dataset than in the inter-host dataset. At the inter-host level, the genetic divergence increased with the scale of the population studied (π nt “FP”<“genotype IV”<“serotype 1”) whereas the proportion of nonsynonymous mutations decreased (d_N_/d_S_ “FP”>“genotype IV”>“serotype 1”), reflecting strong purifying selection pressures.

## Discussion

In this study, DENV-1 evolution was analyzed during two recent outbreaks in FP separated by a four-year period of low-level transmission. Original dynamics of epidemics were revealed in the FP ecosystem. Our results suggest that a significant part of DENV-1 evolution occurred during the 2002–2005 endemic years. Despite evidence for strong negative selection, we report mutations that could reflect viral adaptation, particularly S222T that has been fixed by viral evolution in the envelope glycoprotein. Importantly, we report for the first time a significant correlation between levels of intra-host DENV genetic variability and clinical outcome.

Historically, FP has experienced successive dengue epidemics that involved the four DENV serotypes [12]–[16],[19]. In contrast with most endemic countries and other islands such as those in the Caribbean, where different DENV serotypes circulate, prolonged co-circulation of several serotypes has never been detected in FP. Most Polynesian DENV epidemics were due to the introduction of a new serotype originating either from the Americas, South-East Asia or the Pacific region. Since 2000, serotype 1 has predominated in the South Pacific region and a significant increase in the number of DENV-1 cases has been observed since spring 2006 in several Pacific islands, particularly in FP and in the neighbouring Cook islands [18],[19],[33],[34]. Classically, dengue fever is not believed to be endemic in the Pacific region and outbreaks are usually linked with the importation of a new virus: it has been shown that multiple and repeated introductions of DENV-1 occurred in the Pacific between 2000 and 2003 from a variety of locations in Asia [35].

Accordingly, our first objective was to identify the origin of the DENV-1 strain responsible for the 2001 outbreak in FP. In accordance with a preliminary study [27], phylogenetic analysis based on a large number of either complete polyprotein or E-gene sequences indicates that the most probable source of this epidemic was an Asian strain, as suggested by the close genetic relationship with a strain isolated in Indonesia in 1998. This finding is in contradiction with the hypothesis that the first DENV-1 outbreak observed in the Pacific Ocean in 2000 in Palau (Micronesia) dispersed secondarily to Polynesia and Melanesia [30],[33],[34] and emphasizes the relation between DENV-1 viruses in Asia and those responsible for recent outbreaks in the Pacific [35]. Figure 2 shows that the strain implicated in the Palau outbreak is only distantly related to FP strains and cannot be implicated as the origin of DENV-1 circulation in FP.

Our second objective was to determine whether or not the 2001 and 2006 FP outbreaks followed the model of iterative reintroductions evoked above. Our results indicate that the Polynesian dynamic of DENV-1 is different from that previously described in other Pacific islands such as New Caledonia [35]. Genetic analysis showed that no new introduction of DENV-1 strains occurred in FP after 2001 and that the virus responsible for the 2001 outbreak evolved *in situ* during the following six years. It circulated under a low level endemic mode until its re-emergence as an epidemic virus in 2006. This phenomenon of re-emergence was previously observed in FP in 1964–1969 for DENV-3 (genotype IV), and in 1979–1985 for DENV-4 (genotype II) [13] and thus may constitute an original epidemiological pattern characteristic for Dengue virus evolutionary dynamics in FP. The specific case of DENV-1 circulation between 2001 and 2006 constitutes a unique model of Dengue virus long term evolution in a given ecosystem which we further investigated through the detailed genetic characterization of 181 infected sera sampled during both endemic and epidemic periods.

The complete polyprotein characterization (obtained directly from serum samples) of 12 DENV-1 viruses collected during the 2001–2006 period gave us the opportunity to analyze viral genetic evolution over this 6-year period. Twenty four nonsynonymous mutations were recorded, distributed all along the polyprotein with one third of mutations occurring in the NS2A and NS4B genes and the lowest rate of variation observed in the NS2B-NS3-NS4A region. Notably, the majority of nonsynonymous mutations appeared during the 2002–2005 endemic years, some of these mutations (two that appeared in 2002 and two that appeared in 2005) being conserved in all subsequent sequences (Table 2).

The observation that viral evolution also occurred during periods of endemic transmission was expected, but the extent of the phenomenon deserved further investigation. Accordingly, a detailed analysis of complete E-gene sequences was performed, which allowed to include a much higher number of sequences (93 FP sequences related to the 2002–2005 endemic period, and 83 FP sequences related to the 2001 and 2006 outbreaks). As previously noted in the case of complete polyprotein analysis, synonymous and nonsynonymous mutations were detected not only during the 2001 and 2006 outbreaks but also during the 2002–2005 endemic years. Statistical analysis showed that the number of variable sites (nt and aa) and the percentage of sequence divergence (π nt) were not higher during outbreaks than during endemic periods (Table 3) -and even suggested the opposite. It may appear to be in conflict with conventional thinking since the total virus replicative turnover would be expected to be higher during epidemics, and thus it would be expected that viral genetic variability occurred mainly during the 2001 and 2006 outbreaks. However, the distribution of viral genetic variability between endemic and epidemic periods was considered carefully, since the delineation between endemic and epidemic periods may appear simplistic. For example, although the 2001 outbreak was considered to end in November, the number of confirmed DENV-1 cases reported monthly by the Institut Louis Malardé was still high until May 2002 (data not shown) and this transitional post-epidemic period may have specific characteristics, different from the actual endemic period.

Altogether, it stands out from our analyses that a significant part of Dengue virus evolution occurred during periods of endemic transmission and not only during outbreaks. Moreover, the majority of amino acid changes were observed during the early stages of the endemic period (Figure 4), suggesting adaptation to new specific environmental conditions. This is notably the case for S222T, the most frequent substitution identified in 88 strains, which appeared in August 2002 and was subsequently fixed by viral evolution. This mutation concerns the envelope protein, a major component at the virion surface implicated in the interaction with host cells, membrane fusion and induction of a protective immune response. Residue 222 is localized in domain II which is implicated in the dimerization of the envelope protein at acidic pH preceding membrane fusion and viral entry into the host cell [36]. This mutation is not described in the literature and it is not present in DENV-1 sequences available on GenBank. The absence of relationship with clinical severity of human infection suggests that S222T is not a virulence factor. This mutation was observed in viruses collected in FP at different time points during the 2001–2006 period with an increasing frequency (9% of sequenced strains in 2002, 67% in 2003, 100% in 2004, 2005 and 2006). This mutation in the envelope glycoprotein of FP DENV-1 viruses may be the result of genetic drift but it may be explained by positive selection also. S222T appears to have been fixed rapidly (10 months) which is not suggestive of a simple genetic drift. Its appearance during a period of endemic transmission (August 2002) and its rapid stabilization through time suggest that S222T would confer a selective advantage to the virus and may possibly be associated with adaptation to the mosquito vector.

Another event suggesting possible virus adaptation to the vector is the mutation K363R. This mutation was present in seven strains recovered in FP from March to December 2006. Residue 363 is localized within the “immunoglobulin-like” domain III of the envelope protein which contains regions thought to be important for receptor binding [36]. This residue belongs to a B-cell epitope (293–402) identified in DENV-1 [37]. As DENV infection confers a prolonged type-specific protective immunity, the hypothesis of an immune selection of this variant in humans is unlikely [4],[5]. Rather, K363R may be the consequence of adaptation to the mosquito vector. Importantly, this mutation occurred only in patients originating from Moorea, Raiatea, Tahaa or the Austral archipelago and was not observed in Tahiti where the majority of cases occurred. Since *Aedes (Stegomyia) polynesiensis*, an endemic mosquito specie widespread in most of islands from the Polynesian Triangle connecting Hawaii and Easter Island to New Zealand, is thought to be an important vector of Dengue virus in rural areas [38],[39], whereas *Aedes (Stegomyia) aegypti* is a major vector in urban and sub-urban zones, the K363R mutation may possibly reflect viral adaptation to *Aedes polynesiensis* in FP islands less urbanized than Tahiti.

Although we provide tentative evidence for the existence of a few adaptive mutations during the 2001–2006 period, DENV-1 evolution over this period is globally characterized by strong negative selection, in accordance with previous studies on DENV-2 and DENV-3 evolution [40],[41]. The low d_N_/d_S_ values (0.100 for polyprotein sequences and 0.091 for E-gene sequences) denote purifying selection and may reflect constraints imposed on Dengue virus evolution by the alternating replication of viruses in humans and mosquitoes. Further striking evidence for negative selection is provided by the analysis of genetic variability of DENV-1 at different levels of evolutionary divergence (Table 5): viral diffusion is associated with increasing purifying constraints as illustrated by the decrease in the d_N_/d_S_ ratio measured in intra-host viral populations (d_N_/d_S_ “FP intra-host” = 0.620), in a population of epidemiologically related viruses (d_N_/d_S_ “FP inter-host” = 0.333), in viruses belonging to the same genotype (d_N_/d_S_ “genotype IV” = 0.058) or to the same serotype (d_N_/d_S_ “serotype 1” = 0.045). These results indicate that only a small proportion of nonsynonymous mutations observed at a given level of evolution are likely to persist at a higher time- and space-scale.

Dengue virus, like other RNA viruses, exhibits extensive intra-host genetic diversity [40]–[45]. We analyzed 662 clones from 16 patients infected with DENV-1 in the study period and observed that the structure of intra-host genetic diversity represents an extreme situation in which purifying selective constraints are lower than at higher levels of evolutionary divergence. As noted in a previous study on DENV-2 and DENV-3 [40],[41],[43],[44], most nonsynonymous mutations occurred in single cases (not identified in more distantly related DENV-1) and genome-defective viruses (with stop codons) were identified (3% of clones) in human sera. Similar results were previously reported in a study of 70 clones obtained from four mosquitoes and 220 clones obtained from 13 patients infected with DENV-1 in Myanmar [42]. Defective viruses may interfere with viral evolution but long term transmission of a stop-codon lineage has been described within humans and mosquitoes infected with DENV-1 [42]: complementation mechanisms may occur in host cells coinfected with both functional viruses and defective viruses.

The large number of samples studied here allowed for the first time a comparative analysis of intra-host DENV-1 diversity according to the clinical presentation of the disease. We found that the extent of sequence diversity varied among infected patients. The composition of DENV-1 populations was different in classical (DF) and in severe infections (DHF and DSS). Although intra-host sequence variability was probably overestimated due to *in vitro* artefacts [46], genetic divergence was significantly lower in severe cases than in classical cases. In severe cases, d_N_ and d_S_ values were significantly lower than in classical presentations. In other words, DENV-1 populations were more genetically homogeneous in DHF or DSS cases than in DF cases. In our study, no correlation was found between the level of intra-host genetic diversity and viral load. Moreover, viral loads were not significantly different between the two groups, in a sample of five severe cases and five DF cases. It is therefore not likely that the lower intra-host genetic diversity observed in severe cases would have been influenced by larger amounts of template DNA in amplification reactions (associated with a more rapid saturation of PCR reaction and thus lower error rates).

The mechanisms that lead to different structures of DENV-1 intra-host genetic diversity according to the clinical severity remain undetermined. We do not know if the differences observed are the cause or the consequence of disease severity. Our findings suggest that further analysis of viral variation in both mosquitoes and human samples may in the future shed new light on dengue infection, pathogenesis and the existence of predictive factors of clinical outcome.

## Supporting Information

Figure S1 Details of the phylogenetic tree based on a 1,759 bp region including the E-gene (Figure 2) showing the in situ molecular evolution of DENV-1 in FP from 2001 to 2006. Taxon names of FP sequences correspond to the year of sampling followed by the serum number. In this condensed tree, branch length is not proportional to genetic distance. Numbers on branches represent bootstrap support for each branch.
(0.28 MB TIF)

Figure S2 Phylogenetic tree based on 53 nucleotide sequences of complete coding region of DENV-1 (Neighbor-Joining method, Kimura 2 algorithm). Taxon names of FP sequences correspond to D1.FP/sample number.year (month, geographical origin, clinical presentation). Taxon names of GenBank sequences correspond to D1.country/last two digits of year of isolation and GenBank accession number. In this condensed tree, branch length is not proportional to genetic distance. Numbers on branches represent bootstrap support for each branch.
(0.49 MB TIF)

Table S1 Primers used for DENV-1 amplification and sequencing.
(0.14 MB DOC)

## Abbreviations

aa: amino acid
DENV: dengue virus
DF: dengue fever
DHF: dengue haemorrhagic fever
DSS: dengue shock syndrome
d_N_: ratio of nonsynonymous substitutions per site
d_S_: ratio of synonymous substitutions per site
FP: French Polynesia
nt: nucleotide
RT-PCR: reverse transcription-polymerase chain reaction
π nt: nucleotide sequence divergence (pairwise distance)
