# Supplementary figures and images for: Dengue 1 Diversity and Microevolution, French Polynesia 2001–2006: Connection with Epidemiology and Clinics
Source: PLoS Negl Trop Dis. 2009 Aug 4;3(8):e493. doi: 10.1371/journal.pntd.0000493 (PMC2714178; doi:10.1371/journal.pntd.0000493)

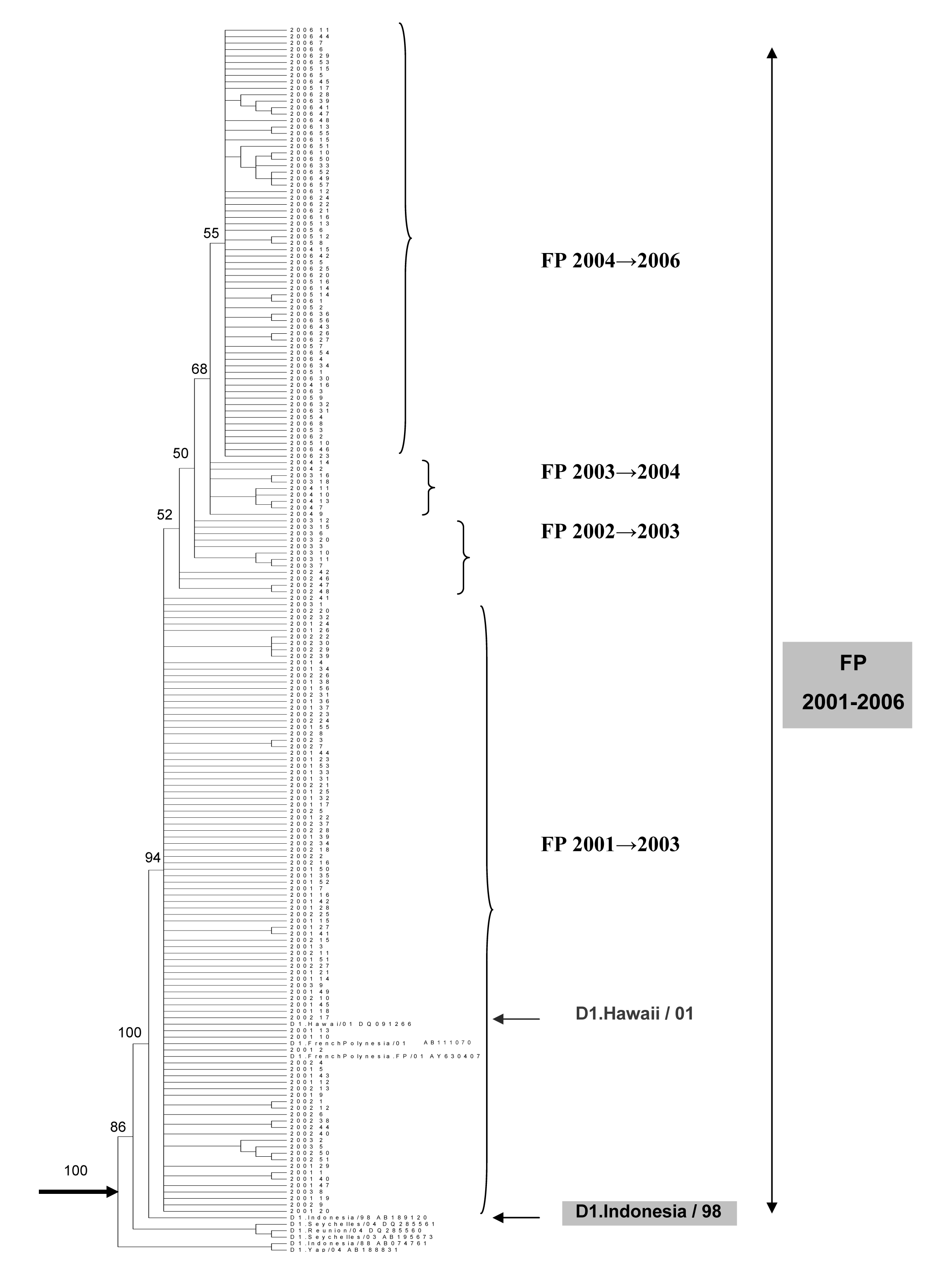

Supplement: Figure S1 — Details of the phylogenetic tree based on a 1,759 bp region including the E-gene (Figure 2) showing the in situ molecular evolution of DENV-1 in FP from 2001 to 2006. Taxon names of FP sequences correspond to the year of sampling followed by the serum number. In this condensed tree, branch length is not proportional to genetic distance. Numbers on branches represent bootstrap support for each branch. (0.28 MB TIF) [file pntd.0000493.s001.tif]

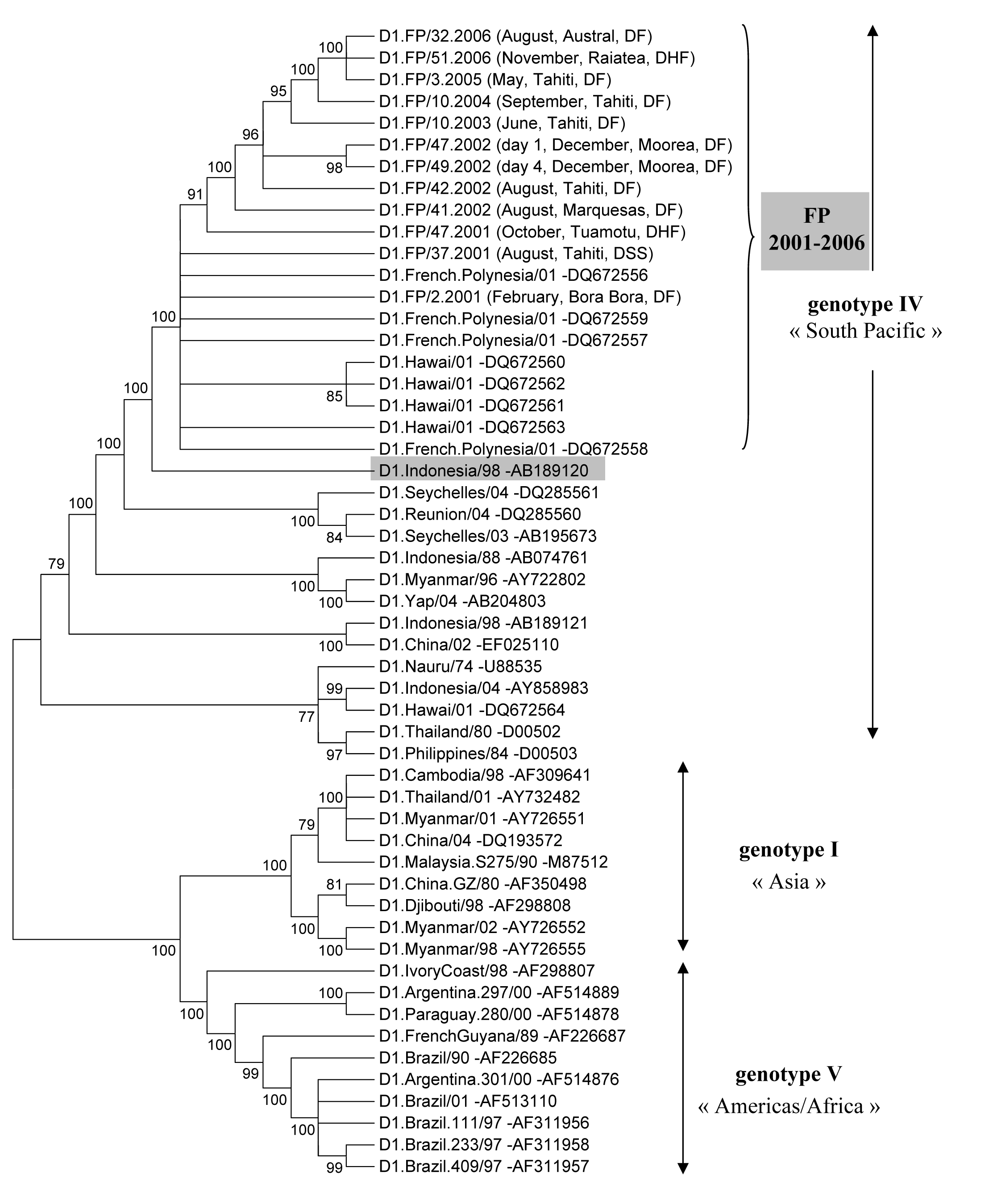

Supplement: Figure S2 — Phylogenetic tree based on 53 nucleotide sequences of complete coding region of DENV-1 (Neighbor-Joining method, Kimura 2 algorithm). Taxon names of FP sequences correspond to D1.FP/sample number.year (month, geographical origin, clinical presentation). Taxon names of GenBank sequences correspond to D1.country/last two digits of year of isolation and GenBank accession number. In this condensed tree, branch length is not proportional to genetic distance. Numbers on branches represent bootstrap support for each branch. (0.49 MB TIF) [file pntd.0000493.s002.tif]
