# Supplementary material for: Dengue 1 Diversity and Microevolution, French Polynesia 2001–2006: Connection with Epidemiology and Clinics
Source: PLoS Negl Trop Dis. 2009 Aug 4;3(8):e493. doi: 10.1371/journal.pntd.0000493 (PMC2714178; doi:10.1371/journal.pntd.0000493)
Supplement: Table S1 — Primers used for DENV-1 amplification and sequencing. (0.14 MB DOC) [file pntd.0000493.s003.doc]

**SUPPORTING INFORMATION**

**Table S1:** Primers used for DENV-1 amplification and sequencing.

| **Studied fragment** | **Primers name** | **Nucleotide sequence**  **5’→ 3’** | **Nucleotide position**^a^ | **Role** |
| --- | --- | --- | --- | --- |
| **12 overlapping fragments (C1-C12) coding for the viral polyprotein** | | | | |
|  | C1F | ATG AWC RAC CAA CGR AAR AAG | 95-115 | RT-PCR and sequencing |
|  | C1R | TWG CTC CYG ABA RTC CTT C | 971-989 | RT-PCR and sequencing |
|  | C2F | ATA GGR ACA TCY ATY ACY CA | 866-885 | RT-PCR and sequencing |
|  | C2R | ATG CTG GGT YTC AGC CAC | 1868-1885 | RT-PCR and sequencing |
|  | C3F | GAA ATY CAR WYG TCW GGA AC | 1739-1758 | RT-PCR and sequencing |
|  | C3R | GCY TTT CCC CAR YTT TTC CA | 2762-2781 | RT-PCR and sequencing |
|  | C4F | ATG TGG AAR CAA ATA TCA AAT GA | 2618-2640 | RT-PCR and sequencing |
|  | C4R | TAB GTY GTT CCC ATS CCC AT | 3677-3696 | RT-PCR and sequencing |
|  | C5F | GTR ATG AGA TCY AGA TGG AG | 3539-3558 | RT-PCR and sequencing |
|  | C5R | CTG CCA AAA RTA CCA CAC RAA | 4484-4504 | RT-PCR and sequencing |
|  | C6F | CAA GAT GAY GGA MCY ATG AA | 4364-4383 | RT-PCR and sequencing |
|  | C6R | TTR TAR TTG GGR ACT CTC AC | 5339-5358 | RT-PCR and sequencing |
|  | C7F | ACA ACA GCA GTR AAR AGY GA | 5252-5271 | RT-PCR and sequencing |
|  | C7R | CAV ATC TCC ACR TCC ATG TT | 6248-6267 | RT-PCR and sequencing |
|  | C8F | CTA TCY TAY AAA GTY GMC TCA | 6158-6178 | RT-PCR and sequencing |
|  | C8R | TTY ACY TGR GAA TAG CAY CC | 7118-7137 | RT-PCR and sequencing |
|  | C9F | GCR GCY ATA YTG ATG GGA C | 7043-7061 | RT-PCR and sequencing |
|  | C9R | TTY ACY AGG TTC CAY CCR TA | 7928-7947 | RT-PCR and sequencing |
|  | C10F | ACC TYG GHT GTG GAA GAG G | 7809-7827 | RT-PCR and sequencing |
|  | C10R | GTR TCW ACY TTC TCT TTA AAC A | 8631-8652 | RT-PCR and sequencing |
|  | C11F | TAY CAY GGA TCA TAT GAG GT | 8483-8502 | RT-PCR and sequencing |
|  | C11R | GTT CCA YTT TTY GCT GGT CT | 9314-9333 | RT-PCR and sequencing |
|  | C12F | ATY ACT GAC ATY ATG GAR CC | 9227-9246 | RT-PCR and sequencing |
|  | C12R | TTR AAT CTC TTC ATT GAT RTC A | 10218-10239 | RT-PCR and sequencing |
|  |  |  |  |  |
| **1,759 nt fragment coding for complete E-gene (935-2419)** ^a^ | | | | |
|  | E1F | AAC AAG ARC YGA RAC RTG GAT GTC | 748-771 | RT-PCR and sequencing |
|  | E2F | ARA TAC AAA RAG TRG ARA CHT GGG C | 792-816 | nested PCR and sequencing |
|  | E3R | TKK GCT GAT CGD ATT CCA CAC AC | 2579-2601 | nested PCR and sequencing |
|  | E4R | YAR TTC ATT TGA TAT TTG YTT CCA CAT | 2619-2644 | RT-PCR and sequencing |
|  | E5F | ACA GAG GCT GGG GYA ATG | 1227-1244 | sequencing |
|  |  |  |  |  |
| **758 nt fragment coding for partial E-gene** | | | | |
|  | Q1F | CTG GAT TGT TCA CCT AGA AC | 1481-1500 | RT-PCR |
|  | Q1R | ACA AAA CTC CAT ACG CAG | 2258-2276 | RT-PCR |

^a^ Nucleotide position according to the D1.Indonésia/88 numbering (GenBank accession number AB074761).

**SUPPORTING INFORMATION**

**Table S1:** Primers used for DENV-1 amplification and sequencing.

| **Studied fragment** | **Primers name** | **Nucleotide sequence**  **5’→ 3’** | **Nucleotide position**^a^ | **Role** |
| --- | --- | --- | --- | --- |
| **12 overlapping fragments (C1-C12) coding for the viral polyprotein** | | | | |
|  | C1F | ATG AWC RAC CAA CGR AAR AAG | 95-115 | RT-PCR and sequencing |
|  | C1R | TWG CTC CYG ABA RTC CTT C | 971-989 | RT-PCR and sequencing |
|  | C2F | ATA GGR ACA TCY ATY ACY CA | 866-885 | RT-PCR and sequencing |
|  | C2R | ATG CTG GGT YTC AGC CAC | 1868-1885 | RT-PCR and sequencing |
|  | C3F | GAA ATY CAR WYG TCW GGA AC | 1739-1758 | RT-PCR and sequencing |
|  | C3R | GCY TTT CCC CAR YTT TTC CA | 2762-2781 | RT-PCR and sequencing |
|  | C4F | ATG TGG AAR CAA ATA TCA AAT GA | 2618-2640 | RT-PCR and sequencing |
|  | C4R | TAB GTY GTT CCC ATS CCC AT | 3677-3696 | RT-PCR and sequencing |
|  | C5F | GTR ATG AGA TCY AGA TGG AG | 3539-3558 | RT-PCR and sequencing |
|  | C5R | CTG CCA AAA RTA CCA CAC RAA | 4484-4504 | RT-PCR and sequencing |
|  | C6F | CAA GAT GAY GGA MCY ATG AA | 4364-4383 | RT-PCR and sequencing |
|  | C6R | TTR TAR TTG GGR ACT CTC AC | 5339-5358 | RT-PCR and sequencing |
|  | C7F | ACA ACA GCA GTR AAR AGY GA | 5252-5271 | RT-PCR and sequencing |
|  | C7R | CAV ATC TCC ACR TCC ATG TT | 6248-6267 | RT-PCR and sequencing |
|  | C8F | CTA TCY TAY AAA GTY GMC TCA | 6158-6178 | RT-PCR and sequencing |
|  | C8R | TTY ACY TGR GAA TAG CAY CC | 7118-7137 | RT-PCR and sequencing |
|  | C9F | GCR GCY ATA YTG ATG GGA C | 7043-7061 | RT-PCR and sequencing |
|  | C9R | TTY ACY AGG TTC CAY CCR TA | 7928-7947 | RT-PCR and sequencing |
|  | C10F | ACC TYG GHT GTG GAA GAG G | 7809-7827 | RT-PCR and sequencing |
|  | C10R | GTR TCW ACY TTC TCT TTA AAC A | 8631-8652 | RT-PCR and sequencing |
|  | C11F | TAY CAY GGA TCA TAT GAG GT | 8483-8502 | RT-PCR and sequencing |
|  | C11R | GTT CCA YTT TTY GCT GGT CT | 9314-9333 | RT-PCR and sequencing |
|  | C12F | ATY ACT GAC ATY ATG GAR CC | 9227-9246 | RT-PCR and sequencing |
|  | C12R | TTR AAT CTC TTC ATT GAT RTC A | 10218-10239 | RT-PCR and sequencing |
|  |  |  |  |  |
| **1.759 nt fragment coding for complete E-gene (935-2419)** ^a^ | | | | |
|  | E1F | AAC AAG ARC YGA RAC RTG GAT GTC | 748-771 | RT-PCR and sequencing |
|  | E2F | ARA TAC AAA RAG TRG ARA CHT GGG C | 792-816 | nested PCR and sequencing |
|  | E3R | TKK GCT GAT CGD ATT CCA CAC AC | 2579-2601 | nested PCR and sequencing |
|  | E4R | YAR TTC ATT TGA TAT TTG YTT CCA CAT | 2619-2644 | RT-PCR and sequencing |
|  | E5F | ACA GAG GCT GGG GYA ATG | 1227-1244 | sequencing |
|  |  |  |  |  |
| **758 nt fragment coding for partial E-gene** | | | | |
|  | Q1F | CTG GAT TGT TCA CCT AGA AC | 1481-1500 | RT-PCR |
|  | Q1R | ACA AAA CTC CAT ACG CAG | 2258-2276 | RT-PCR |

^a^ Nucleotide position according to the D1.Indonésia/88 numbering (GenBank accession number AB074761).
